# Recent Advances and Future Directions in Extracorporeal Carbon Dioxide Removal

**DOI:** 10.3390/jcm14010012

**Published:** 2024-12-24

**Authors:** Tomás Lamas, Susana M. Fernandes, Francesco Vasques, Christian Karagiannidis, Luigi Camporota, Nicholas Barrett

**Affiliations:** 1 ICU Department at Hospital Egas Moniz, ULSLO, 1349-019 Lisbon, Portugal; 2 ICU Department at CUF Tejo, 1350-352 Lisbon, Portugal; 3 Faculdade de Medicina, Universidade de Lisboa, 1649-028 Lisbon, Portugal; susanamfernandes@medicina.ulisboa.pt; 4 Serviço de Medicina Intensiva, ULS Santa Maria, 1649-035 Lisbon, Portugal; 5 Department of Adult Critical Care, Guy’s and St Thomas’ NHS Foundation Trust, King’s Health Partners, London SE1 9RT, UK; francesco.vasques@gstt.nhs.uk (F.V.); luigi.camporota@gstt.nhs.uk (L.C.); nicholas.barrett@gstt.nhs.uk (N.B.); 6 Division of Centre of Human Applied Physiological Sciences, King’s College London, London WC2R 2LS, UK; 7 Department of Pneumology and Critical Care Medicine, ARDS and ECMO Centre, Cologne-Merheim Hospital, 51109 Cologne, Germany; christian.karagiannidis@uni-wh.de

**Keywords:** extra-corporeal CO_2_ removal (ECCO_2_R), acute respiratory distress syndrome (ARDS), haemolysis, thrombosis

## Abstract

Extracorporeal carbon dioxide removal (ECCO_2_R) is an emerging technique designed to reduce carbon dioxide (CO_2_) levels in venous blood while enabling lung-protective ventilation or alleviating the work of breathing. Unlike high-flow extracorporeal membrane oxygenation (ECMO), ECCO_2_R operates at lower blood flows (0.4–1.5 L/min), making it less invasive, with smaller cannulas and simpler devices. Despite encouraging results in controlling respiratory acidosis, its broader adoption is hindered by complications, including haemolysis, thrombosis, and bleeding. Technological advances, including enhanced membrane design, gas exchange efficiency, and anticoagulation strategies, are essential to improving safety and efficacy. Innovations such as wearable prototypes that adapt CO_2_ removal to patient activity and catheter-based systems for lower blood flow are expanding the potential applications of ECCO_2_R, including as a bridge-to-lung transplantation and in outpatient settings. Promising experimental approaches include respiratory dialysis, carbonic anhydrase-coated membranes, and electrodialysis to maximise CO_2_ removal. Further research is needed to optimise device performance, develop cost-effective systems, and establish standardised protocols for safe clinical implementation. As the technology matures, integration with artificial intelligence (AI) and machine learning may personalise therapy, improving outcomes. Ongoing clinical trials will be pivotal in addressing these challenges, ultimately enhancing the role of ECCO_2_R in critical care and its accessibility across healthcare settings.

## 1. Introduction

Extracorporeal carbon dioxide removal (ECCO_2_R) is a technique designed to remove carbon dioxide from the venous blood, thereby reducing the intensity of mechanical ventilation and the patient’s work of breathing. Unlike high-flow extracorporeal membrane oxygenation (ECMO), ECCO_2_R does not provide oxygenation, as it operates at lower blood flows ranging from 0.4 up to 1.5 L/min, depending on the device. The lower blood flow reduces the procedure’s invasiveness, allowing smaller cannulae and simpler extracorporeal devices. To refine this lung-support technology, various ECCO_2_R configurations and techniques have been explored in benchtop and animal models.

Although there is a specific interest in using ECCO_2_R to improve the management of acute respiratory distress syndrome (ARDS) and chronic obstructive pulmonary disease (COPD), ECCO_2_R shows potential for other applications, including serving as a bridge to lung transplantation and enabling long-term CO_2_ management in outpatient settings. However, ECCO_2_R faces barriers such as haemolysis, thrombosis, and bleeding, which limit its clinical adoption. This review highlights recent advancements in ECCO_2_R technology, including device configurations, CO_2_ removal efficiency, and optimised anticoagulation strategies. We explore the physiological impacts of ECCO_2_R systems studied through animal models and mock circulation setups to evaluate safety and performance. We also explore key research priorities, including improving gas exchange membranes, refining blood flow and anticoagulation protocols to reduce complications, and investigating innovative approaches such as membrane acidification and hybrid devices.

## 2. Clinical Evidence for ECCO_2_R

ECCO_2_R has been investigated for its role in managing acute respiratory distress syndrome (ARDS) and acute hypercapnic respiratory failure, particularly acute exacerbations of chronic obstructive pulmonary disease (AECOPD). The earliest model of ECCO_2_R utilised a pumpless arterio-venous (AV) approach, diverting a portion of the patient’s cardiac output through the device [1] via a femoral arterio-venous circuit. Several case reports and case series documented the use of pumpless AV ECCO2R [2,3,4,5,6,7,8,9,10,11,12] and demonstrated their ability to decrease arterial CO_2_ and resolve respiratory acidosis. However, complications associated with the use of AV ECCO_2_R were considerable, including bleeding (18–47%), thrombosis (0–20%), and limb ischaemia (4.5–22%) [2,12].

Pumped ECCO_2_R systems were later developed, enabling a veno-venous (VV) approach, typically using a double-lumen cannula placed in the femoral or jugular vein [13]. Animal studies consistently show that VV ECCO_2_R can effectively control respiratory acidosis [14,15,16,17,18]. Similarly, uncontrolled clinical studies demonstrated similar results in patients with ARDS and AECOPD [12,16,19,20,21,22,23,24]. However, a retrospective propensity-matched case–control study using VV ECCO_2_R in COPD found no difference in outcome with ECCO_2_R use [25]. Recently, VV ECCO_2_R has been successfully integrated with renal replacement therapy platforms in patients with ARDS [26,27] and AECOPD [28]. Case series have also reported reduced intubation rate in AECOPD and decreased respiratory rate when used alone or with non-invasive mechanical ventilation [29,30,31]. 

In ARDS management, the delivery of lung-protective ventilation—reducing driving pressure, optimising PEEP, limiting plateau pressure and tidal volume—is hindered by significant respiratory acidosis and the risk of acute cor pulmonale. Trials, such as the SUPERNOVA study, investigated using ECCO_2_R to achieve ultra-low tidal volumes of 3–4 mL/kg in mechanically ventilated ARDS patients, demonstrating feasibility and safety in controlling arterial CO_2_ [26]. The UK-based REST trial, the first randomised controlled trial in this field, compared standard tidal volume ventilation (6–8 mL/kg) to ultra-low tidal volume ventilation (3–4 mL/kg) using ECCO_2_R. While the trial achieved significant separation between groups in tidal volume and maintained comparable PaCO_2_ and pH, it showed no difference in 90-day mortality. 

The ECCO_2_R group encountered significantly higher complication rates, including bleeding and intracranial haemorrhage. These complications negated any potential benefits of lung rest, as they necessitated increased use of mandatory mechanical ventilation and neuromuscular blockade. Serious adverse events, such as intracranial haemorrhage, ultimately led to the early termination of the trial due to safety concerns [32].

For AECOPD, non-invasive ventilation (NIV) remains the gold standard. However, ECCO_2_R has been explored as an option for patients who fail or cannot tolerate NIV. Trials have shown faster resolution of respiratory acidosis and work of breathing with ECCO_2_R, though they were not powered to assess mortality. Other studies have evaluated ECCO_2_R for preventing intubation or enabling early extubation, finding no differences in ventilator-free days or mortality, with higher mortality in the NIV group when ECCO_2_R was added [33]. There is limited evidence on the use of ECCO_2_R as a bridge to lung transplantation in patients requiring invasive mechanical ventilation. In these situations, extracorporeal gas exchange—whether via ECMO or ECCO_2_R—enables patients to remain awake, breathing spontaneously, and mobile. This strategy has proven effective for individuals with terminal fibrosis and severe hypercapnia, with ECCO_2_R successfully facilitating lung transplantation in such cases [34,35,36].

Despite mixed results, interest in ECCO_2_R persists, particularly regarding the optimal blood flow rate for clinical benefit. Evidence suggests that higher-flow devices (500–1000 mL/min) provide superior CO_2_ clearance compared to lower-flow systems (<500 mL/min) [37,38]. All the RCTs to date have used devices with a maximum blood flow below 500 mL/min. Future trials should investigate the potential of higher blood flow ECCO_2_R devices to improve clinical outcomes.

## 3. Key Areas of Future Research

The future of ECCO_2_R remains challenging due to the clinical outcomes observed so far and the significant complications associated with current devices. Priority should be given to addressing technical issues such as haemolysis, thrombosis, and bleeding. Research must also define optimal parameters for blood flow, sweep gas efficiency, and monitoring while developing methods to compare different devices for practical application. Experimental studies using appropriate circuits and biological models are essential to translate findings into clinical practice. Ultimately, well-designed clinical trials addressing patient-centred outcomes will be crucial in determining this technology’s role in critically ill patients.

### 3.1. Haemolysis

Haemolysis is a significant complication of extracorporeal support and is independently associated with higher mortality [39,40]. A key determinant of haemolysis is the shear stress caused by blood circulating through artificial circuits. This shear rate and stress arise from artificial surfaces and continuous flow patterns [41]. Factors such as catheter size and type, flow rates, pump design (roller or centrifugal), lung membrane characteristics, anticoagulation methods (coating and systemic), and ECCO_2_R settings all influence haemolysis rates and system performance.

Haemolysis is best detected by measuring plasma free Hb (PFHb), the most reliable marker of RBC injury and breakdown [42,43]. Evidence suggests that haemolysis increases non-linearly with reducing blood flows, as prolonged exposure to artificial surfaces amplifies the effective stress on blood components [37,44].

When the haemoglobin scavenging capacity of haptoglobin is exceeded, free haemoglobin dimers circulate in the bloodstream, releasing free haem [45]. Free haemoglobin depletes nitric oxide (NO) by converting it into nitrate, impairing NO’s regulatory role in vascular smooth muscle tone. The resulting NO depletion leads to potent vasoconstriction and increased systemic and pulmonary vascular resistance [46,47,48]. Additionally, NO depletion disrupts platelet and endothelial function, enhancing platelet aggregation and thrombus formation mediated by the von Willebrand factor [49,50,51].

Free haemoglobin initiates a cycle of inflammation that culminates in a procoagulant state and thrombus formation. This process exacerbates haemolysis, depletes clotting factors, and leads to thrombocytopenia and impaired platelet function [52], which may result in an acquired von Willebrand syndrome [53,54].

To address this frequent complication, we require improved devices and a greater clinical understanding of how device usage impacts haemolysis.

### 3.2. Thrombosis and Bleeding

**Figure 1:**
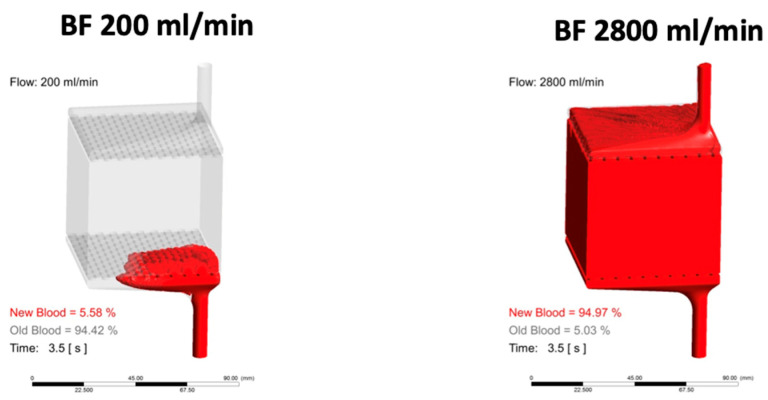
Example of different blood washouts depending on high and low blood flows in membrane oxygenator with impact on anticoagulation requirements to prevent membrane clotting. Pictures kindly provided by F. Hesselmann and R. Borchardt, Aachen, Germany.

Anticoagulation is a critical concern in extracorporeal circulation technology, given that anticoagulation and the impact of shear stress on coagulation proteins may lead to increased bleeding risk [21,25,29,32,55,56,57]. On the other hand, thrombosis is responsible for locally impaired flow conditions in cannulas, pumps, and membrane lungs (Figure 1), which lead to mechanical stress and haemolysis. The coagulation cascade is activated when whole blood comes into contact with an artificial surface, exacerbated by slow blood flow (Figure 1). Relative slow blood flow occurs in low-shear circuit parts, such as tubing connectors, with increased thrombin generation [58,59] and minimal clot initiators required to trigger clot formation [60]. The artificial membrane oxygenator and the centrifugal head pump are the two main circuit components affected by higher mechanical stress and risk of thrombus formation. Centrifugal head pump thrombosis is usually associated with acute and severe haemolysis [40]. Membrane thrombosis reduces gas exchange impacting device efficiency. 

Contrary to expectations, low blood flow rates (1–1.5 L/min) are associated with a significantly higher risk of haemolysis (3.2 to 6.6 times greater Haemolysis Index) compared to higher blood flow (~4 L/min). In low-flow systems (<1.5 L/min), citrate anticoagulation has been shown to outperform heparin by causing less haemolysis and platelet loss [61]. Citrate better preserves red blood cell density, membrane stability, and deformability, reducing haemolysis over three days of storage [62]. In animal studies, Cardenas et al. demonstrated no thrombus formation and haemolysis at different low-flow levels (500 mL/min, 800 mL/min, 1000 mL/min) using citrate anticoagulation in 24 h. Although higher blood flows may reduce calcium chelation efficiency due to elevated ionised calcium levels, this did not correlate with thrombus formation. When comparing citrate to heparin anticoagulation at electronic microscopy, analysis of membrane oxygenator fibres revealed cellular and fibrin adhesion on heparin anticoagulation even after 6 h of anticoagulation [63]. Despite these theoretical advantages, heparin remains the most commonly used anticoagulant in clinical ECCO_2_R due to concerns about citrate toxicity. 

A significant complication of citrate anticoagulation in animal studies was progressive hypocalcaemia, even with calcium supplementation, due to the absence of a haemofilter to remove excess citrate. This increased systemic citrate load, heightening the risk of citrate toxicity [63,64,65]. Further research is needed to enhance the safety profile of citrate anticoagulation for clinical ECCO_2_R.

An emerging alternative to sodium citrate is citric acid anticoagulation, which provides a triple effect: calcium chelation, platelet inhibition, and a regional anticoagulant effect from an acidic environment [66]. Citric acid has also enhanced CO_2_ removal by blood acidification at the artificial membrane [67]. However, hepatic clearance of citrate in humans is limited [68], which reduces the maximum blood flow of ECCO_2_R to 150 mL/min, which can be offset by an increase in CO_2_ removal efficiency driven by the acidification of the blood [66,69,70,71]. 

Other alternatives to heparin, such as nafamostat mesylate, bivalirudin, and argatroban, are being considered, especially for patients at high risk of bleeding or heparin-induced thrombocytopenia (HITT). These alternatives offer advantages regarding bleeding risks and drug monitoring but are limited by high costs and short half-lives.

## 4. CO_2_ Removal Rate Performance

### 4.1. Cannula

ECCO_2_R typically employs smaller cannulas (13–14 Fr) than the larger cannulas used in higher-flow ECMO. Venous access is generally achieved via the right internal jugular or femoral veins, with vessel puncture performed under ultrasound guidance. While the smaller cannulas offer advantages such as reduced invasiveness, their use presents several challenges that can impact both efficacy and patient safety. A key issue is maintaining adequate blood flow through the smaller cannulas. A smaller diameter cannula with higher flow rates increases shear stress, which may reduce CO_2_ removal efficiency due to insufficient flow, ultimately limiting the therapy’s effectiveness. Multiple single-lumen catheters can replace double-lumen catheters. This involves using separate access points in the jugular and femoral veins to achieve uninterrupted blood flow of approximately 450 mL/min with minimal recirculation [72]. Another relevant factor for ECCO_2_R efficiency is the minimisation of blood recirculation related to catheters. Catheter recirculation was related to catheter type and brand, site of placement, catheter length, and time on the current catheter and was measured via ultrasound dilution technique [73]. Catheters placed in the internal jugular or subclavian veins, with the tip near the right atrium, minimise recirculation, making them more effective for ECCO_2_R. In contrast, femoral catheters have higher recirculation rates and are less suitable for this therapy.

### 4.2. Pumps, Membranes and Circuits

#### 4.2.1. Pumps

Conventionally, ECCO_2_R uses roller, centrifugal or diagonal, electric or electromagnetic pumps. The roller pumps used in ECCO_2_R systems were developed for renal replacement therapy (RRT) [74,75,76] or centrifugal pumps used for high-flow extracorporeal membrane oxygenation (ECMO) devices, with few systems explicitly designed for ECCO_2_R [14,29,77]. However, RRT devices driven by roller pumps are limited in blood flow rates (usually up to 500 mL/min), limiting the CO_2_ removal performance. Standard ECMO centrifugal pumps running below 2 L/min cause increasing shear stress, leading to haemolysis [44].

Potential new pump technologies are on the horizon, for example, an innovative membrane and pump integrated to operate at very low flow (250 mL/min) using six rotating impellers inside a cylindrical membrane configuration with a total surface area of 0.42 m^2^. Blood moves from inside to outside through membrane fibres, allowing up to 75 mL/min CO_2_ removal rate with a haemolysis level comparable to standard CO_2_ removal devices [78].

#### 4.2.2. Membranes

**Figure 2:**
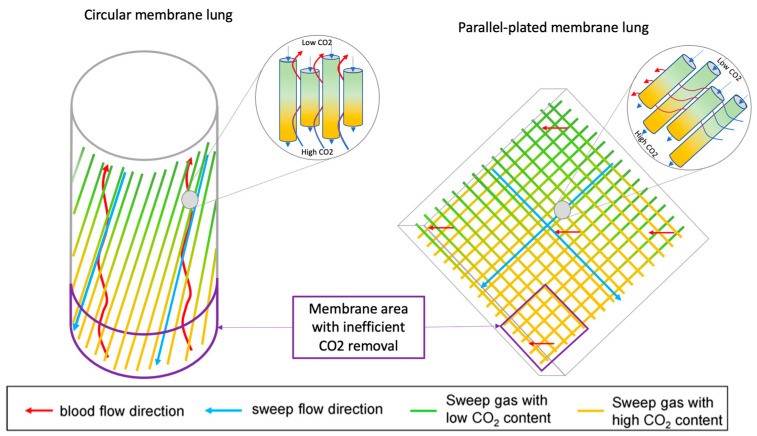
Fibre arrangement in parallel plate and circular membrane lungs (ML). In the circular ML, blood flow is nearly antiparallel to the gas fibres and flow. In the lower part of the ML, where blood enters, blood encounters sweep gas with the highest CO_2_ concentration. Since CO_2_ removal depends on the diffusion gradient between the gas and blood—small in this region—CO_2_ clearance is inefficient here. In the parallel-plate MLs, blood flows perpendicularly to the gas fibres. Similarly, at the lower part of the ML, where blood interacts with gas containing the highest CO_2_ levels, CO_2_ removal is less effective. With permission from Schwärzel et al. [80].

Three major factors determine the amount of gas crossing membranes: the diffusion gradient, the membrane–blood contact time, and the characteristics of the membrane diffusion. The CO_2_ diffusion gradient is determined by the CO_2_ content of the blood and the air passing through the membrane lung, as well as the speed of the airflow. Membrane–blood contact time is determined by membrane geometry. Modern membrane lungs achieve adequate gas exchange with 1 to 3 m^2^ surface areas (Table 1). To allow any benchmarking between different membrane sizes and shapes, the VCO_2_ of each membrane and device should be standardised to enable comparison and assessment of performance. Each company should declare its index by membrane surface area (i.e., mL/min/m^2^) [78]. This way, clinicians can choose the most efficient membrane and encourage companies to develop better membranes. The study of Hospach and co-authors demonstrated two different membrane areas (Prismalung+ 0.8 m^2^, 190 mm length, blood volume 86 mL and Eurosets 1.35 m^2^, 210 mm length, blood volume 190 mL), obtaining equivalent VCO_2_. Removing the heat exchanger fibre at “Prismalung+” allows the streamlining of blood and gas fibres and reduces the potential for pooling and low flow within the device, giving the lowest blood volume ratio to the membrane surface. This reduced area size is an advantage due to less priming volume and anticoagulation, but it has the drawback of higher resistance and pressure drop [79]. Related to carbon dioxide removal efficiency between circular and parallel-plated gas exchange membranes at different sweep gas and blood flows, Schwärzel et al. found that parallel-plated membranes achieved overall highest CO_2_ removal rates under medium and high sweep gas flow rates in opposite to better performance at the lowest gas flow rate in circular membranes (Figure 2). The circular membranes may be more adequate for low gas flow (0.5 L/min) due to reduced shunting associated with their fibre orientation. However, this low gas flow is not relevant for everyday clinical practice. 

**Table 1:** Comparison of available ECCO_2_R in the market based on the pump (pumpless, roller, and centrifugal) and membrane function (gas and/or fluid CO_2_ removal). The table summarises the key characteristics of roller and centrifugal pumps, focusing on parameters such as blood flow limits, priming volume, membrane surface area, membrane material, CO_2_ extraction efficiency, VCO_2_ monitor and circuit life [19,27,81,82,83,84,85].

Pump Type	Pumpless	Roller								Centrifugal			
Membrane Function	Gas CO_2_ Removal	Gas CO_2_ Removal	Gas CO_2_ Removal/Hemofilter						CO_2_ Fluid Removal	Gas CO_2_ Removal			
Blood flow (mL/min)	500–4500	100–450	30–450	200–400			200–800	200–500	100–400	350–500	500–1000	100–7000	2500–7000
Vascular access	Arterio-venous	Veno-Venous											
Cannula size	15 Fr	13–14 Fr	13 Fr	13.5 Fr	13.5 Fr	13.5 Fr	15.5 Fr	13.5 Fr	13–14 Fr	15.5 Fr	18–19 Fr	18–26 Fr	Drainage 25–29 Fr
Cannula configuration	Arterial and venous	Double lumen	Double-lumen	Double-lumen	Double-lumen	Double-lumen	Double-lumen	Double-lumen	Double lumen	Double-lumen	Single or Double-lumen	Single or double lumen	Reinjection: 17–21 Fr
Priming volume (mL)	175	220	200–500	140–160	155–190	100	130	130	200 mL (albumin)	200–300	250–350	605	300–500
Membrane position related to Hemofilter	-	-	Pre	Post	Post	Post	Pre	Pre	-	-	-	-	-
Membrane surface (m^2^)	1.3	1.8	0.33-1.35	0.32	0.8	1.35	1.8	1.8	none	0.59	0.65	1.9	1.8
Membrane/coating	PMP	PMP/PC	PMP	PMP/Hep	PMP/PC	PMP/PC	PMP/PC	PMP/PC	(n.a.)	PMP/Sil+Hep	PMP	PMP	PMP/PC
CO_2_ extraction (% of initial value)	50–60	20–35	n.a.	<25	>50	79–80	76–78	80–90	25–50	25	50	>50	>50
VCO_2_ Monitor	No	Yes	No	No	No	No	Yes	No	No	Yes	No	No	No
Validated circuit life		5 days	5 days	3 days	3 days	3 days	5 days	3 days	24h	7 days		30 days	30 days
Brand	iLA Activve^®^ 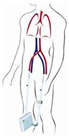	ProLung 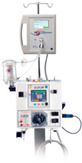	Decap Smart 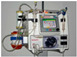	Prismalung membrane 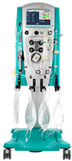	Prismalung Plus 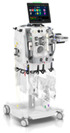	multiECCO_2_R Eurosets membrane 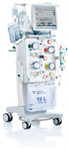	CO_2_ Reset, Eurosets 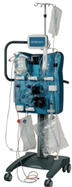	multiECCO_2_R Eurosets membrane, 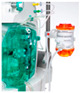	ADVOS 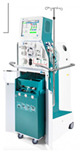	ALung Hemolung^®^RAS 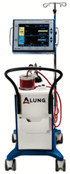	MiniLung^®^ Novalung 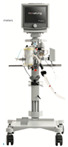	XLung^®^ Novalung 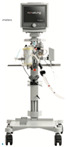	Maquet Cardiohelp^®^ 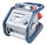
Manufacturer, city	Xenios AG, Fresenius Medical Care, Radeberg, Germany	Estor, Milan Italy	Medica Group, Mirandola, Italy	Baxter International Inc., Deerfield, IL, USA	Baxter International Inc., Deerfield, IL, USA	Eurosets Membrane, Fresenius Medical Care, Gröbenzell, Germany	Eurosets S.r.l., Medolla, Italy	Eurosets membrane, B. Braun SE, Hessen, Germany	ADVITOS GmbH, Munich, Germany	LivaNova, London, UK	Xenios, Fresenius Medical Care, Radeberg, Germany	Xenios, Fresenius Medical Care, Radeberg, Germany	Getinge, Gothenburg, Sweden

PMP, Poly-4-methyl-1-pentene; PC, polycarbonate; Sil + Hep, siloxane + heparin.

##### Materials

Early fibres were constructed with microporous polypropylene. Micropores create microscopic blood–gas interfaces, allowing efficient gas exchange and causing plasma leaks. Recently, non-microporous poly-4-methyl-1-pentene (PMP) has been used; it provides superior gas exchange, better biocompatibility, and is less susceptible to plasma leak [86,87,88]. The gas exchange has been improved by arranging fibres into a complex mat and running blood on the outside [89]. This arrangement allows perpendicular blood flow to the fibres, enhancing mass transfer by reducing the diffusion path length compared to parallel flow. 

##### Novel Surfaces for ECCO_2_R

New materials and surface coatings are designed to reduce the inflammatory and coagulation responses triggered by artificial surfaces. Bioactive coatings, such as heparin-based and nitric oxide (NO)-releasing materials, mimic natural endothelium and prevent blood clotting. Membranes may include a nitric oxide (NO)-eluting NO-eluting ECCO_2_R system, tubing with NO-catalysing surface coating or NO gas (80 ppm) delivered into the membrane that inhibits platelet activation and aggregation to minimise thrombosis during extracorporeal CO_2_ removal (ECCO_2_R) [90].

Endothelialisation of surfaces is an emerging approach, with efforts to create artificial materials that mimic the human endothelial layer, potentially reducing the need for systemic anticoagulation and preventing thrombosis. Further innovations focus on developing fully biocompatible materials to avoid bleeding and clotting complications without compromising oxygenation efficiency, raising the possibility for long-term respiratory support in chronic lung failure [91]. These include bioactive hollow fibre membranes (HFMs) coated with carbonic anhydrase (CA). CA immobilisation on HFMs increases the conversion of bicarbonate into CO_2_, improving removal efficiency by creating a steeper diffusion gradient at the membrane surface. The CA-coated membranes also improved haemocompatibility, reducing platelet adhesion by 95% [92]. Acidic sweep gas containing sulfur dioxide (SO_2_), in combination with carbonic anhydrase (CA)-coated hollow fibre membranes (HFMs), can significantly enhance CO_2_ removal from blood. This technique offers the potential for developing more efficient ECCO_2_R devices. The findings could lead to smaller, less invasive respiratory support systems for patients with acute respiratory failure [93].

Innovative therapies, such as intravascular gas exchange devices, hold promise for lung support in acute respiratory conditions both in and outside the hospital. They also have long-term potential for managing chronic lung diseases while preserving patient mobility through continuous ECCO_2_R. Despite their appeal, this technology has yet to reach clinical use. Only one device, the IVOX catheter, has advanced to human clinical trials but has yet to receive FDA approval. Technical challenges have hindered their progress, including optimising gas exchange within the vascular space and ensuring safety. Advancements in design may eventually offer a less invasive alternative to ECMO for managing acute respiratory failure [94].

##### Measuring Device VCO_2_ to Assess Membrane Performance

When comparing devices and evaluating efficiency, it is crucial to understand how CO_2_ is measured to estimate carbon dioxide transfer across the artificial membrane. This can be carried out by assessing trans-membrane CO_2_ content differences using whole blood CO_2_ content using the Douglas equation [95] or measuring the partial pressure of CO_2_ in the effluent gas using infrared CO_2_ sensors [96]. In both approaches, normalising to a standardised inlet PCO_2_ is essential to define the device’s operating range and enable meaningful efficiency comparisons.

One of the concerns with effluent gas VCO_2_ measurement is the need for more independent validation of VCO_2_. However, this has been performed with one device, little is known about the accuracy of the devices in which VCO_2_ is measured with an infrared sensor measurement (Table 1) [79,97]. To improve comparative evaluations, the VCO_2_ of each membrane and device should be standardised to inlet CO_2_ and blood flow for consistent comparison and performance assessment. Manufacturers should ideally report their device’s index as VCO_2_ per membrane surface area (e.g., mL/min/m^2^) [78].

### 4.3. Combined CO_2_ Removal (“Lung Dialysis”) with Renal Support

**Figure 3:**
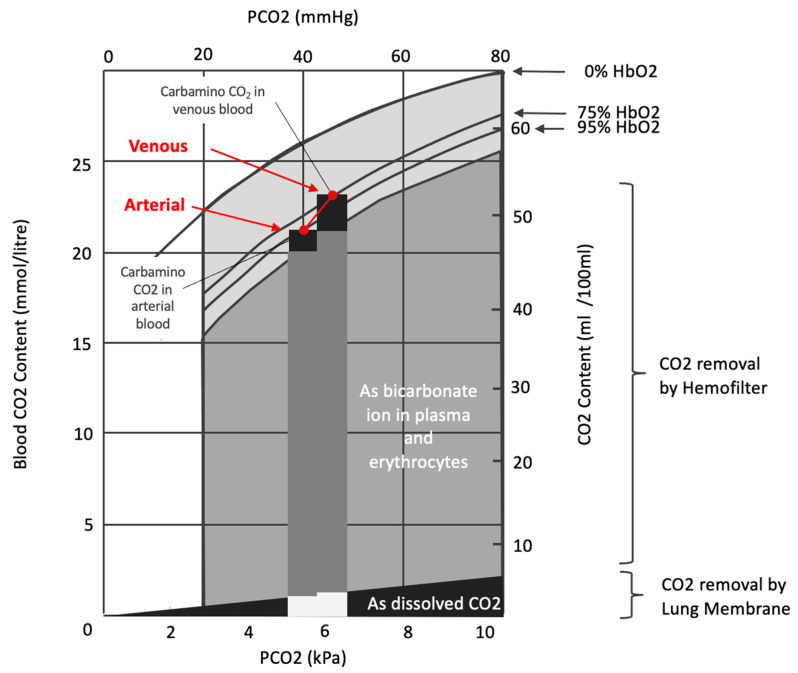
The proportion of carbamino form contribution from arterial CO_2_ content to venous CO_2_ content is significantly higher than the other forms of CO_2_ content due to the Haldane effect (increasing haemoglobin affinity to CO_2_ in lower oxygen concentration). The CO_2_ removal of the gaseous phase of CO_2_ represents only a small fraction of total CO_2_ content. The bicarbonate removal using zero bicarbonate dialytic solution through haemofilter could contribute to significantly higher CO_2_ removal of total CO_2_ content. However, the blood buffer must be replaced by other natural buffers to maintain the acid–base equilibrium.

Other strategies for enhancing CO_2_ removal from the blood focus on methods targeting bicarbonate, as around 90% of CO_2_ in the blood is transported as bicarbonate (Figure 3). The gas exchange membrane can be isolated or combined with a “renal” membrane (haemofilter) to achieve this. Research has shown that combining extracorporeal CO_2_ removal (ECCO_2_R) with CRRT effectively reduces arterial CO_2_ (PaCO_2_), improves pH, and stabilises haemodynamics in patients with acute respiratory distress syndrome (ARDS) and renal failure. Despite these benefits, mortality rates in critically ill patients remain high, especially in the most severe cases [98]. Integrating a hollow-fibre gas exchanger into CRRT platforms is relatively simple, and the gas exchange membrane can be placed before or after the haemofilter. Some data suggest that the CO_2_ removal rate tends to be higher when the membrane is before the haemofilter. However, the clinical impact of this remains unknown [74]. An alternative strategy is integrating gas and fluid fibres in parallel using a shared circuit [99].

The optimal dialysis solution to use in combined devices remains to be discovered. Commonly available bicarbonate-based solutions increase total blood CO_2_ content, which is problematic when high dialysis rates are required for solute clearance. Alternative solutions allow the simultaneous removal of H^+^ and bicarbonate, maintaining acid–base balance whilst allowing CO_2_ removal rates of up to 160 mL/min [85]. Several approaches seek to manipulate regional pH to alter bicarbonate or CO_2_ clearance. Alkalinisation methods with bicarbonate-free solutions which enhance bicarbonate removal are being investigated in animal models [100]. However, these methods may prove impractical for clinical use due to acid–base derangements, haemolysis, cardiac arrhythmias, and depletion of micronutrients, even though several approaches to replace bicarbonate have been attempted [100,101]. Respiratory electrodialysis, which increases CO_2_ partial pressure in the blood through regional acidification, has been shown to remove approximately 50% of the total CO_2_ metabolic production, offering a promising strategy for efficient CO_2_ removal. These varied strategies illustrate ongoing efforts to develop more effective means of extracorporeal CO_2_ removal, particularly for critically ill patients with multiple organ failure [102].

## 5. Conclusions

The future of ECCO_2_R holds significant promise. However, two actions need to occur in parallel. First, the technology needs to be improved to reduce the impact on blood and coagulation whilst simultaneously increasing the efficiency of CO_2_ removal. Second, the population who may benefit and the timing of ECCO_2_R need to be better defined. ECCO_2_R is being evaluated for patients with ARDS and COPD, but other groups, including patients bridging to transplant, may benefit.

Advances in membrane technology and gas exchange efficiency have the potential to minimise the size and blood flow requirements of ECCOR systems, improving their safety and accessibility. These improvements could lead to the development of smaller, catheter-based systems that are less invasive and more practical for broader clinical use [100]. Developing new anticoagulation strategies for ECCO_2_R is crucial, given the delicate balance between preventing clot formation within the circuit and minimising the risk of bleeding complications in patients. Artificial intelligence (AI) and machine learning could act similarly to the respiratory centres, allowing automated changes in CO_2_ clearance based on changes in blood CO_2_ and pH [103]. Ultimately, miniaturisation, the development of closed feedback loops, reduced impact on blood/coagulation, and efficiency improvements will benefit the ICU population and may also allow the development of wearable devices that act as destination or prolonged bridging therapies [104].

Clinical trials are ongoing and will determine ECCO_2_R’s efficacy, safety, and best practices. The effectiveness of ECCO_2_R could be investigated in patients with moderate ARDS presenting with hypercapnia and respiratory acidosis, focusing on outcomes such as survival, haemodynamic changes, and duration of ventilatory support. The selection of the right patients who are more likely to benefit and the mechanical ventilation during ECCO_2_R need to be established. Comparative studies with VV-ECMO are crucial to determine whether ECCO_2_R offers advantages in terms of safety, cost, and patient outcomes.

For COPD patients, particularly those at risk of NIV failure or requiring invasive mechanical ventilation, ECCO_2_R has potential benefits in reducing respiratory rate and alleviating overinflation. However, its efficacy remains largely unproven. Trials should examine whether ECCO_2_R reduces intubation rates, facilitates early weaning, and improves overall outcomes compared to standard care. Furthermore, systematic studies are needed to evaluate high-extraction ECCO_2_R systems versus other technologies to establish optimal device configurations and their impact on clinical effectiveness and complications.

In patients awaiting lung transplantation, maintaining mobility and spontaneous breathing is paramount. Research should prioritise less invasive ECCO_2_R systems that avoid femoral cannulation, which may hinder ambulation. Trials should compare less invasive approaches, such as upper-body cannulation or double-lumen cannulas, to assess their impact on mobility, safety, and bridging success rates. Additionally, studies should explore the role of low-flow ECCO_2_R systems versus VV-ECMO, focusing on differences in complication profiles and ease of management.

For patients with terminal lung fibrosis and severe hypercapnia, ECCO_2_R may represent the only viable option for supporting respiratory failure and enabling progression to transplantation. Trials should evaluate various ECCO_2_R technologies, particularly less invasive systems, to optimise patient mobility and stability during the pre-transplant period.

Overall, ECCO_2_R’s future looks bright, with ongoing innovation and research likely to expand its clinical applications and affect critical care practice.
